# Systolic hypertension, caesarean duration and prenatal visits as predictors of maternal near miss in Peru

**DOI:** 10.1093/inthealth/ihaf045

**Published:** 2025-04-29

**Authors:** Estefany Alejandra Cutipa Vásquez Melgar, Rodrigo Jesús Flores Palacios

**Affiliations:** Faculty of Health Sciences Private University of Tacna, Peru; Intensive and Intermediate Care Unit, Hospital III Daniel Alcides Carrión – EsSalud, Tacna, Peru; Faculty of Health Sciences, National Jorge Basadre Grohmann University, Tacna, Peru

**Keywords:** caesarean section, cohort studies, maternal health, maternal health service, precipitating factors

## Abstract

**Background:**

To identify the predictive factors of maternal near miss in caesarean patients in the obstetrics and gynaecology service at Hospital III Daniel Alcides Carrión, Tacna, Peru.

**Methods:**

A retrospective cohort study was conducted from 1 January 2022 to 31 December 2023. Preoperative, intraoperative and postoperative clinical and laboratory characteristics of caesarean patients hospitalized in the obstetrics and gynaecology service were analysed. Cox proportional hazards regression was used to identify predictors.

**Results:**

We identified 264 caesarean patients, of which 49 experienced maternal near miss. The mean age was 32.81±5.13 y, the median number of prenatal visits was 7 (interquartile range [IQR] 6–9) and the median gestational age was 39 weeks (IQR 37.5–40). Identified predictive factors for maternal near miss were systolic blood pressure ≥140 mmHg before caesarean (adjusted hazard ratio [aHR] 2.20), duration of the caesarean (aHR 1.02) and number of prenatal visits (aHR 0.90).

**Conclusions:**

The findings suggest that systolic hypertension before caesarean delivery, caesarean duration and number of prenatal visits are significant predictors of maternal near miss. These results underscore the importance of early prenatal care, monitoring blood pressure levels and optimizing surgical duration to improve maternal outcomes. Future research should focus on the implementation of targeted interventions based on these predictors to reduce maternal morbidity and improve health policies in low-resource settings.

## Introduction

Maternal near miss (MNM) is a significant public health concern that affects a considerable number of women during pregnancy and childbirth globally, posing substantial risks not only to patients’ lives, but also to healthcare systems.^1,2^ This condition is often associated with severe maternal morbidity, which is preventable with proper healthcare intervention.^3^ The incidence of caesarean deliveries has increased globally, often viewed as a safer alternative in complex situations, particularly in the context of MNM, and due to medico-legal factors or patient preferences in the absence of specific indications for vaginal delivery.^4^

This increase in caesarean sections may introduce additional risks, as it adds surgical complications to the baseline maternal health conditions. International studies have identified several triggers for MNM, such as sepsis, severe systemic infections, pre-eclampsia/eclampsia and the use of blood products.^5^ The relationship between these factors and MNM has been extensively studied in various healthcare settings worldwide, highlighting the need for more data in different geographic regions.^6^

In low-resource settings, like Peru, women experiencing MNM tend to be younger (ages 20–34 y), often from rural areas and more likely to be nulliparous or multiparous.^7^ Factors such as more than six prenatal visits and adequate interpregnancy intervals have been observed, but there remains limited analytical research focusing on the predictors of MNM.^8^ In Peru, despite some descriptive studies, there is a notable gap in analytical research examining the factors that contribute to MNM, particularly among caesarean patients.^7,8^

Caesarean deliveries are associated with increased risks of complications, including haemorrhage, infection and long-term morbidity, which may exacerbate the incidence of MNM.^4^ Given the increasing number of caesarean deliveries and the persistence of maternal mortality as a significant issue in the country,^9^ it is essential to identify the predictive factors of MNM in this population.

This study aims to describe MNM and identify the predictive factors of MNM in caesarean patients at the Hospital III Daniel Alcides Carrión (HDAC) in Tacna from 1 January 2022 to 31 December 2023. By focusing on caesarean patients, who are at a heightened risk of complications, the study intends to provide valuable insights that could guide future clinical practices and policies to reduce the burden of MNM.

## Methods

### Study design and population

We conducted an observational, retrospective cohort study, including all caesarean patients hospitalized in the obstetrics and gynaecology service of HDAC, the only social security hospital in the Tacna region of Peru, from 1 January 2022 to 31 December 2023. The main exposure was a low number of prenatal visits, which we hypothesized to be associated with a higher risk of MNM. Electronic medical records were reviewed through the Smart Health Service.^10,11^

Tacna is an urban city, with 87.3% of its population residing in urban areas.^12^ The region is characterized by its high educational indicators, with 85.7% of individuals >15 y of age having completed at least a secondary education,^13^ and one of the lowest poverty rates in the country, ranging from 11.8% to 14.6%.^14^ The region ranks third in the Human Development Index with a value of 0.6425.^15^ A total of 92.3% of women of reproductive age have at least a secondary education and the female literacy rate is 97.8%.^16^

A total of 868 caesarean deliveries were performed, from which two patients <18 y of age and one patient who was referred to another institution were excluded. To determine the sample size, we used a sample size calculation formula based on differences in proportions,^17^ considering an exposure ratio of 0.31 (history of prenatal visits) and assuming an MNM rate of 58.8% in patients without prenatal visits, compared with 19.9% in those who had at least one prenatal visit.^18^ With these data and a 95% confidence level, we obtained a sample of 264 caesarean patients hospitalized in the obstetrics and gynaecology service of HDAC. The statistical power of our sample was calculated to be 99.94%, due to the large difference in expected proportions between the compared groups.

### Study variables

The dependent variable was MNM, recorded as dichotomous (yes/no). It was considered affirmative if the patient presented at least one of the following conditions after caesarean delivery:^19^ cardiovascular failure, respiratory failure, renal failure, haematological disorder, hepatic failure, neurological disorder, uterine dysfunction or admission to the intensive care unit (ICU).

The main independent variable was the number of prenatal visits. Other independent variables included age, parity, history of abortion, history of caesarean delivery, interpregnancy interval, gestational age, presence of urinary tract infection in the third trimester, hypertension and diabetes mellitus.

The main independent variable was the number of prenatal visits. Additional independent variables included maternal characteristics such as age, parity, history of abortion and caesarean delivery, interpregnancy interval, gestational age, urinary tract infection in the third trimester, hypertension and diabetes mellitus. Preoperative physiological parameters encompassed heart rate, blood pressure (BP; systolic ≥140 mmHg, diastolic ≥90 mmHg),^20^ oxygen saturation and laboratory values, including haemoglobin (<11 g/dl, indicating anaemia),^21^ platelets, leucocytes, creatinine and glucose (>100 mg/dl, indicating impaired regulation).^22^ Surgical and postoperative characteristics comprised anaesthesia type (epidural, spinal, general), caesarean duration in minutes, amniotic fluid appearance (clear, yellow, meconial, bloody), oxytocin administration (none, 20 IU, 30 IU), intravenous iron administration and postoperative indicators including haemoglobin (<9 g/dl),^23^ time to caesarean section (>2 d) and hospital stay duration (>3 d).^24,25^ These cut-off values and clinical parameters are based on previous studies and evidence supporting their relevance as thresholds for identifying obstetric risk conditions.

The study did not include sociodemographic variables such as maternal education, wealth quintile or geographic location, as these data were poorly recorded in the medical records, with a significant number of missing values. Additionally, the population studied is relatively homogeneous in these aspects, which reduces the potential impact of such variables on the study outcomes.

Follow-up of cases was performed during their hospital stay, from admission to the obstetrics and gynaecology department until the presentation of MNM or hospital discharge.

### Bias control

To mitigate selection bias, we included all patients who met the inclusion criteria during the study period. Multivariate analysis included adjustments for age, comorbidities and other factors that could act as confounding variables, such as the number of prenatal visits and a history of hypertension or diabetes. Measurement bias was minimized through the use of standardized electronic medical records.

Some variables, such as glucose and creatinine, had missing data in a small proportion (<10%) of patients. However, it was observed that these missing values predominantly came from stable patients who did not require these tests due to the absence of severe complications. Since these variables were not significant in the multivariate analyses, a complete case analysis was performed. This approach was deemed suitable, as the missing data did not affect the key predictors of MNM. However, it is acknowledged that this strategy could introduce limitations if the missing variables were critical for identifying predictors.

### Data analysis

The data were analysed using Stata version 14 (StataCorp, College Station, TX, USA) for statistical analysis. Quantitative variables were evaluated for normality using the Shapiro–Wilk test. Median and interquartile range (IQR) or mean and standard deviation (SD) were reported. Categorical variables were presented in terms of frequencies and percentages.

Bivariate analysis was conducted using the χ^2^ test or Fisher's exact test for categorical variables and the Mann–Whitney U test or Student's t-test for continuous variables, depending on the distribution. In the multivariate analysis, Cox proportional hazards regression was used, adjusting for variables that had a p-value <0.20 in the bivariate analysis. The Cox regression model was selected to evaluate the time to the occurrence of MNM, due to the retrospective cohort nature of the study. In the adjusted analysis, variables with a p-value <0.20 and a variance inflation factor <10 were considered. Final results were reported as adjusted hazard ratios, with a p-value <0.05 and a 95% confidence interval (CI). Graphs for data visualization were created using R Studio 2024 (Posit Software, Boston, MA, USA).

Finally, the survival of patients undergoing caesarean section was described using the Kaplan–Meier method. The logrank test was employed to assess differences between the survival functions.

## Results

We analysed data from 264 patients, of which 49 (18.56%) experienced MNM. The main causes of MNM were hypertensive disorders of pregnancy (69.39%) and postpartum haemorrhage (20.41%). The main criteria for classifying MNM were uterine dysfunction and admission to an ICU (both at 24.49%).

**Figure 1.**
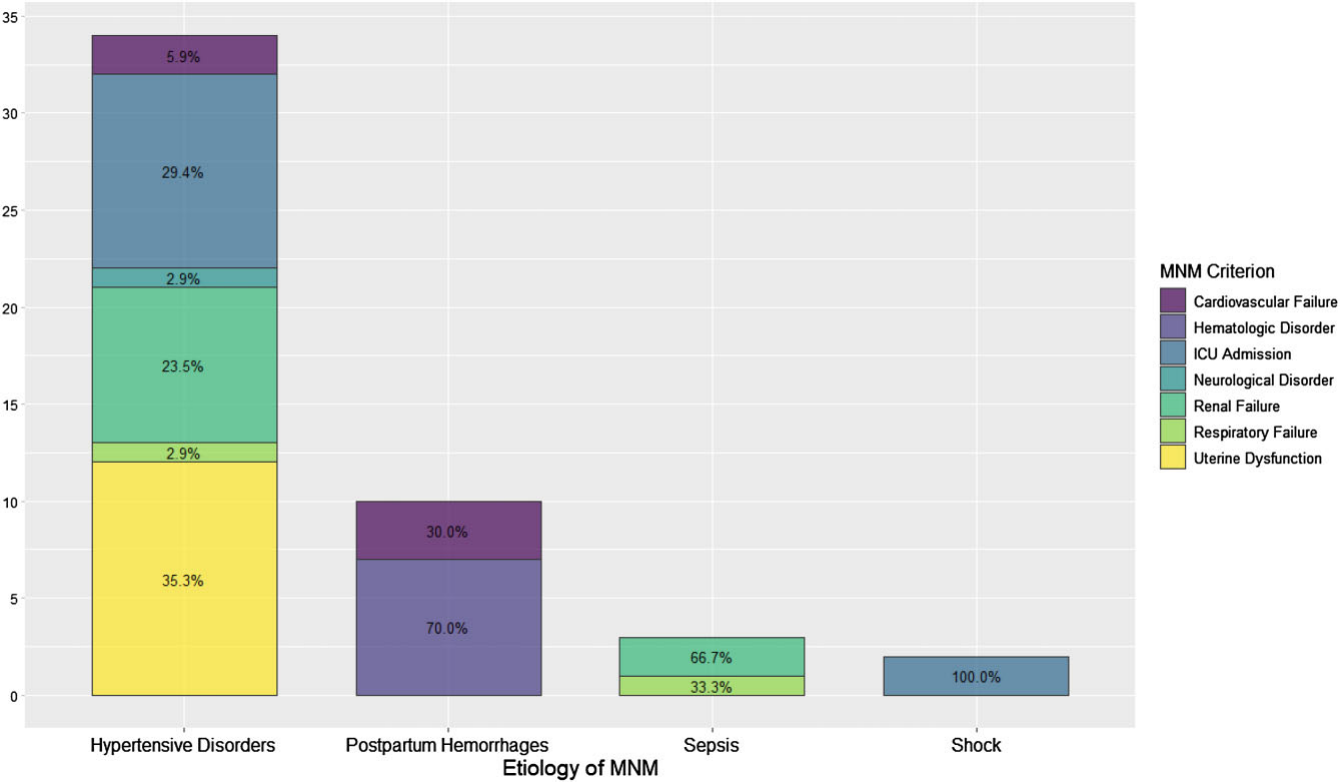
Distribution of MNM criteria by aetiology at HDAC. The stacked bar chart displays the distribution of maternal near miss (MNM) criteria across various etiologies. The bar heights represent the absolute number of cases (n), while the percentages indicate the proportion of each criterion within each etiology. The colors correspond to different MNM criteria. ICU: Intensive Care Unit.

Figure 1 shows the distribution of MNM cases by aetiology and main criterion. Absolute values are indicated on the y-axis, while the percentages of each main criterion are displayed in the labels within the bars. Hypertensive disorders accounted for 34 cases, postpartum haemorrhage for 10 cases, sepsis for 3 cases and shock for 2 cases. The total number of MNM cases was 49. This visualization allows for observation of the prevalence of each aetiology and the proportion of associated criteria.

The main characteristics of caesarean patients hospitalized in the obstetrics department are presented in Table 1. The mean age was 32.81±5.13 y, the majority were nulliparous (39.39%), 45.83% had a history of at least one abortion and 43.94% had a history of one or more previous caesarean sections. The median number of prenatal visits was 7 (IQR 6–9). The gestational age was 39 weeks (IQR 37.5–40). Among the patient's, 15.15% had a urinary tract infection in the third trimester, 4.92% had hypertension and 4.55% had diabetes mellitus.

**Table 1.** Clinical-obstetric, perioperative and temporal characteristics according to MNM of patients hospitalized at HDAC.

		MNM		
Characteristics	All patients (N=264)	No (n=215)	Yes (n=49)	p-Value
**Clinical-obstetric characteristics**				
Age (years), median±SD	32.81±5.13	32.66±5.10	33.51±5.23	0.293^a^
Parity, n (%)				0.268^b^
Nulliparous	104 (39.39)	80 (76.92)	24 (23.08)	
Primiparous	92 (24.85)	79 (85.87)	13 (14.13)	
Multiparous	68 (25.76)	56 (82.35)	12 (17.65)	
History of abortion, n (%)				0.863^b^
No	143 (54.17)	117 (81.82)	26 (18.18)	
Yes	121 (45.83)	98 (80.99)	23 (19.01)	
History of caesarean section, n (%)				0.037^b^
No	148 (56.06)	114 (77.03)	34 (22.97)	
Yes	116 (43.94)	101 (87.07)	15 (12.93)	
Prenatal visits, n (IQR)	7 (6–9)	8 (6–9)	7 (5–7)	0.004^c^
Interpregnancy interval				0.471^b^
Normal	152 (57.58)	121 (79.61)	31 (20.39)	
Short	28 (10.61)	25 (89.29)	3 (10.71)	
Long	84 (31.82)	69 (82.14)	15 (17.86)	
Gestational age (weeks), median (IQR)	39 (37.5–40)	39.1 (38–40.2)	37.4 (36–39.1)	<0.001^c^
UTI in the third trimester, n (%)				0.851^b^
No	224 (84.85)	182 (81.25)	42 (18.75)	
Yes	40 (15.15)	33 (82.50)	7 (17.50)	
Arterial hypertension, n (%)				<0.001^b^
No	251 (95.08)	212 (84.46)	39 (15.54)	
Yes	13 (4.92)	3 (23.08)	10 (76.92)	
Diabetes mellitus, n (%)				0.557^b^
No	252 (95.45)	206 (81.75)	46 (18.25)	
Yes	12 (4.55)	9 (75.00)	3 (25.00)	
**Preoperative characteristics**				
Heart rate (bpm), median (IQR)	80 (75.5–82)	80 (74–80)	82 (80–90)	<0.001^c^
Systolic BP ≥140 mmHg, n (%)				
No	225 (85.23)	204 (90.67)	21 (9.33)	<0.001^b^
Yes	39 (14.77)	11 (28.21)	28 (71.79)	
Diastolic BP ≥90 mmHg, n (%)				
No	233 (88.26)	205 (87.98)	28 (12.02)	<0.001^b^
Yes	31 (11.74)	10 (32.26)	21 (67.74)	
Oxygen saturation (%), median (IQR)	98 (97–99)	98 (97–99)	97 (97–98)	0.002^c^
Preoperative Hb <11 g/dl, n (%)				
No	196 (74.24)	164 (83.67)	32 (16.33)	0.113^b^
Yes	68 (25.76)	51 (75.00)	17 (25.00)	
Platelets (×10^3^ cells/dl), median (IQR)	249 (210 -293.5)	250 (210–294)	236 (206–292)	0.451^c^
Leucocytes (×10^3^ cells/dl), median (IQR)	8.97 (7.65–10.49)	8.77 (7.57–10.46)	9.18(7.82–10.72)	0.452^c^
Creatinine (mg/dl), median (IQR)^d^	0.73(0.65–0.80)	0.72(0.64– 0.80)	0.76(0.68–0.84)	0.045^c^
Glucose >100 mg/dl, n (%)^e^				0.020^b^
No	194 (81.33)	162 (83.51)	32 (16.49)	
Yes	44 (18.67)	30 (68.18)	14 (31.82)	
**Intraoperative characteristics**				
Anaesthesia type, n (%)				0.209^b^
Epidural	149 (56.44)	126 (84.56)	23 (15.44)	
Spinal	99 (37.50)	78 (78.79)	21 (21.21)	
General	16 (6.06)	11 (68.75)	5 (31.25)	
Caesarean time (min), median (IQR)	55 (45–63.5)	53 (44–60)	58 (49–70)	0.031^c^
Amniotic fluid appearance, n (%)				
Clear	194 (73.48)	157 (80.93)	37 (19.07)	0.657^b^
Yellow	17 (6.44)	13 (76.47)	4 (23.53)	
Meconial	49 (18.56)	42 (85.71)	7 (14.29)	
Bloody	4 (1.52)	3 (75.00)	1 (25.00)	
**Postoperative characteristics**				
Oxytocin use (UI), n (%)				0.223^b^
None	3 (1.14)	3 (100.00)	0 (0.00)	
20 UI	112 (42.42)	96 (85.71)	16 (14.29)	
30 UI	149 (56.44)	116 (77.85)	33(22.15)	
Postoperative Hb <9 g/dl, n (%)				0.020^b^
No	231 (87.50)	193 (83.55)	38 (16.45)	
Yes	33 (12.50)	22 (66.67)	11 (33.33)	
Intravenous iron use, n (%)				0.081^b^
No	230 (87.12)	191 (83.04)	39 (16.96)	
Yes	34 (12.88)	24 (70.59)	10 (29.41)	
**Temporal characteristics**				
Time to caesarean section >2 d, n (%)				<0.001^b^
No	245 (92.80)	206 (84.08)	39 (15.92)	
Yes	19 (7.20)	9 (47.37)	10 (52.63)	
Time >2 d in hospital after caesarean section, n (%)				<0.001^b^
No	165 (62.50)	151 (91.52)	14 (8.48)	
Yes	190 (37.50)	64 (64.65)	35 (35.35)	
Total hospital stay >3 d, n (%)				<0.001^b^
No	191 (72.35)	173 (90.58)	18 (9.42)	
Yes	73 (27.65)	42 (57.53)	31 (42.47)	
Hospital stay duration (days), median (IQR)	3 (2–4)	3 (2–3)	4 (3–6)	<0.001^b^

UTI: urinary tract infection; Hb: haemoglobin; UI: international units.

^a^Student's t-test.

^b^χ^2^.

^c^Mann–Whitney U test.

^d^Not recorded in 16 patients.

^e^Not recorded in 26 patients.

In the bivariate analysis, we found that patients with MNM had a lower number of prenatal visits (median 7 vs 8; p=0.004) and a lower gestational age (median 37.4 vs 39.1 weeks; p<0.001). Additionally, patients with MNM had a higher prevalence of hypertension (76.92% vs 15.54%; p<0.001), systolic BP ≥140 mmHg (71.79% vs 9.33%; p<0.001) and diastolic BP ≥90 mmHg (67.74% vs 12.02%; p<0.001).

Among the preoperative characteristics, we observed a higher heart rate (median 82 vs 80 bpm; p<0.001) and higher creatinine levels (median 0.76 vs 0.72 mg/dl; p=0.045) in patients with MNM. Postoperatively, these patients more frequently had haemoglobin levels <9 g/dl (33.33% vs 16.45%; p=0.020) and longer hospital stays after caesarean delivery (>2 d; p<0.001). Moreover, the total length of hospitalization was longer in this group (median 4 vs 3 d; p<0.001).

### Predictive factors for maternal near miss in caesarean patients

In the subsequent Cox regression analysis (Table 2), we identified that a higher number of prenatal visits was associated with a 10% reduction in the adjusted risk of MNM (aHR 0.90 [95% CI 0.81 to 0.99], p=0.039).

**Table 2.** Predictive factors for MNM in caesarean patients: Cox regression analysis.

Factors	MNM, cHR (95% CI)	p-Value^a^	MNM, aHR (95% CI)	p-Value^a^
History of cesarean section				
No	Ref.			
Yes	1.29 (0.68 to 2.46)	0.426	–	–
Number of prenatal visits	0.93 (0.84 to 1.02)	0.118	0.90 (0.81 to 0.99)	0.039
Gestational age	0.99 (0.91 to 1.08)	0.828	–	–
Arterial hypertension				
No	Ref.			
Yes	1.17 (0.55 to 2.48)	0.678	–	–
Preoperative haemoglobin <11 g/dl				
No	Ref.			
Yes	1.03 (0.57 to 1.88)	0.912	–	–
Creatinine	0.81 (0.31 to 2.16)	0.679	–	–
Glucose >100 mg/dl				
No	Ref.		Ref.	
Yes	2.45 (1.28 to 4.69)	0.007	1.98 (0.95 to 4.13)	0.070
Heart rate	1.00 (0.98 to 1.02)	0.950	–	–
Systolic BP ≥140 mmHg				
No	Ref.		Ref.	
Yes	2.27 (1.19 to 4.34)	0.013	2.20 (1.10 to 4.37)	0.025
Diastolic BP ≥90 mmHg				
No	Ref.			
Yes	1.47 (0.76 to 2.84)	0.258	–	–
Oxygen saturation	1.01 (0.82 to 1.25)	0.939	–	–
Cesarean time (min.)	1.01 (1.00 to 1.02)	0.139	1.02 (1.00 to 1.03)	0.018
Postoperative haemoglobin <9 g/dl				
No	Ref.			
Yes	0.98 (0.49 to 1.96)	0.945	–	–
Intravenous iron use				
No	Ref.			
Yes	1.16 (0.57 to 2.36)	0.676	–	–
Time to caesarean section >2 d				
No	Ref.		Ref.	
Yes	0.55 (0.26 to 1.18)	0.123	0.71 (0.32 to 1.58)	0.405
Time >2 d in hospital after caesarean section				
No	Ref.			
Yes	0.87 (0.42 to 1.79)	0.702	–	–

^a^Cox regression.

Although elevated glucose (>100 mg/dl) initially showed a 150% increase in the crude hazard ratio of complications (cHR 2.45 [95% CI 1.28 to 4.69], p=0.007), this effect was not maintained after adjusting for other variables (aHR 1.98 [95% CI 0.95 to 4.13], p=0.070), suggesting that the association might be influenced by other factors.

Elevated systolic BP (≥ 140 mmHg) was associated with a 120% increase in the adjusted risk of complications (aHR 2.20 [95% CI 1.10 to 4.37], p=0.025).

Furthermore, each increment in the duration of the caesarean was associated with a 2% increase in the adjusted risk of complications (aHR 1.02 [95% CI 1.00 to 1.03], p=0.018).

**Figure 2.**
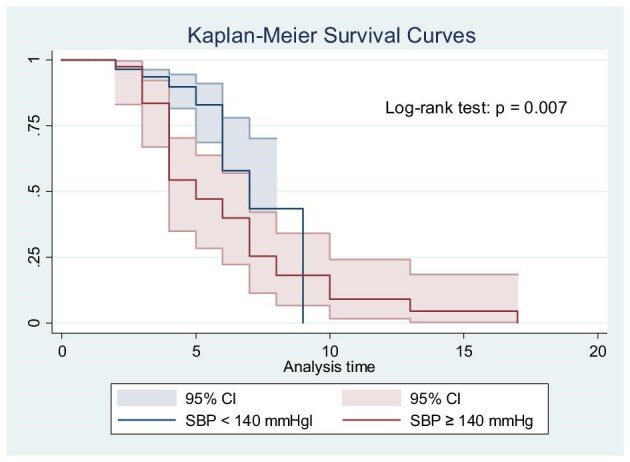
Kaplan–Meier survival curve depicting the cumulative risk of MNM over time, stratified by systolic BP.

The Kaplan–Meier analysis was performed to evaluate the survival function based on systolic BP (≥140 mmHg), as this was the only categorical variable found to be statistically significant in the multivariate analysis. The logrank test revealed a significant difference in the survival functions, with a p-value of 0.007 (Figure 2). Continuous variables, such as caesarean time and prenatal visits, were not included in this analysis, as they were not categorical and were not suitable for the Kaplan–Meier method.

## Discussion

In our study, the incidence of MNM was 18.56%. The multivariate analysis using Cox regression identified three key predictive factors: each additional prenatal visit reduced the risk of MNM by 10%, systolic BP ≥140 mmHg before caesarean increased the risk 2.2 times and each additional minute in caesarean duration increased the risk by 2%.

The global weighted prevalence of MNM was 18.67/1000 (95% CI 16.28–21.06), much lower than that reported in our study. This is likely because the previous World Health Organization definition had stricter severity criteria,^26^ while the current criteria allow any organ dysfunction to be considered a near-miss event, thus increasing sensitivity for early recognition of this condition.

Hypertensive disorders of pregnancy were the leading cause of MNM, affecting 69.39% of patients, followed by postpartum haemorrhage (20.41%), sepsis (6.12%) and shock (4.08%). These findings are in line with previous studies, such as one conducted in Africa, where pregnancy-induced hypertension and obstetric haemorrhage were the leading causes of MNM.^27^ This persistence in causes reinforces what had been observed in our country until 2016, where hypertensive disorders, obstetric sepsis^28^ and pre- and postpartum haemorrhages^29,30^ were also common causes of MNM, similar to global observations.^30^

Women who attended fewer prenatal visits had a higher risk of MNM, with a 10% reduction in risk for each additional prenatal visit. This result is supported by studies conducted in Ethiopia,^27^ India^31^ and Morocco,^32^ which also found that lack of prenatal care increases the risk of MNM. Prenatal visits are essential for monitoring both the mother and the foetus,^33^ and fewer visits may hinder the detection of anomalies or predispositions to complications, such as maternal obesity,^34^ elevated BP^35^ or urinary tract infections.^36^ Thus strategies to improve access to and utilization of prenatal care in low-resource settings are critical. Promoting a higher number of prenatal visits is crucial to prevent maternal morbidity and mortality, particularly in underserved populations. In clinical practice, this could involve increasing the availability of prenatal appointments, providing education on the importance of early and regular visits and reducing financial or logistical barriers to access.

Another relevant finding was the impact of systolic BP. Women with systolic BP ≥140 mmHg before caesarean had 2.2 times higher odds of developing MNM. This contrasts with the study by Asaye in Ethiopia,^37^ where the odds were 5.3 times higher. The difference may be due to continuous monitoring and effective measures adopted in our region, such as the use of magnesium sulphate and specialized care in ICUs.

To improve management of hypertensive disorders, early screening for high BP and timely interventions, along with close monitoring, should be prioritized in clinical settings to reduce the risk of MNM.

Caesarean duration also emerged as an important factor. In patients with MNM, the average caesarean duration was 58 min, compared with 53 min in those without MNM. For every additional minute in caesarean duration, the risk of MNM increased by approximately 2%. This finding is consistent with the study by Rottenstreich et al.,^38^ which found a relationship between prolonged surgical times and a higher risk of MNM, as well as the need for blood transfusions and longer hospital stays.

A longer surgical time for caesarean indicates an intraoperative complication, leading to higher morbidity.^39^ To reduce caesarean duration and its associated risks, clinical protocols that focus on optimizing surgical techniques, improving team coordination and reducing delays during the procedure could be beneficial. Furthermore, ensuring that teams are well trained to handle potential complications more efficiently may help minimize the duration of the surgery.

### Limitations

The main limitation of our study lies in its retrospective cohort design, which may have led to the omission of confounding variables not recorded in the medical records, such as alcohol consumption, smoking or key inflammatory markers like C-reactive protein and procalcitonin. Additionally, the focus on a single healthcare institution limits the generalizability of the results, posing a significant restriction when extrapolating findings to other populations or settings. Despite these limitations, our study provides valuable evidence regarding the predictive factors of MNM in caesarean patients. These findings lay a foundation for future multicentre studies that could offer broader insights and inform the development of specific prevention programs for similar populations.

## Conclusions

Our study found that a higher number of prenatal visits is associated with a lower risk of MNM, highlighting its importance as a predictive factor. Systolic BP ≥140 mmHg was also identified as a significant predictive factor for MNM, underscoring the need for early detection and management of hypertension. Moreover, prolonged caesarean duration increases the risk of MNM, with even smaller increments in the duration of the procedure associated with higher risk.

These findings emphasize the importance of prenatal monitoring and appropriate management during caesarean delivery to prevent MNM. To improve the utilization of prenatal care in low-resource clinical settings, strategies such as enhancing access to prenatal appointments through mobile health technologies, community-based education on the importance of regular visits and the integration of risk factor screenings (such as hypertension and anaemia) at the primary care level could be considered. These interventions would help ensure that at-risk women receive timely care, potentially reducing the incidence of MNM.

## Data Availability

Data will be made available upon request.
